# Cancer

**Published:** 1874-06

**Authors:** 


### CANCER.

No facts have been observed which authorize the conclusion that cancer is an inherited disease; it is always a local disease, begins at some point in the body and disseminates itself until the whole constitution is impregnated with it and every fibre of the body becomes cancerous, that is, contains more or less of the cancerous principle, just as a poisonous bite beginning at a pin point spreads its baleful effects until the whole system comes within the deadly shadow of it. Thus it is that real cancer can never be eradicated by cutting it out; the whole blood is cancerous, and must develop itself somewhere; just like a root of clover—cut it out of the ground, it will grow no more at that spot, but there are tendrils to the main root which have shot out a foot or more away, which will shoot up in the form of half a dozen clover plants, when the parent stock has been removed.

There is a spurious cancer, apparently like the real, and which is often cured with a variety of poultices, and the persons who do this set themselves up as real cancer curers, and draw their patients from points thousands of miles away. The better plan in all cases, perhaps, is to rely on the opinion of some resident, educated physician.

Although cancerous persons do not propagate the disease, they do beget consumptive children. Consumption is always the result of debility, of a want of general good health, whatever may be the causes of the same; but cancer often attacks persons who have vigorous health. Cancer is terribly painful, with a slow march to inevitable death, with a horrible odor during the whole sickness. There is an impression on many minds that the finger nails are poisonous to a sore, from the frequent observation of the fact that sores conveniently situated for "picking"

get well slowly, if at all; the reason is that if a sore is disturbed frequently, as on the lip or about the nose, it begins to lose the power of healing; and nature, seemingly angry in being so often thwarted in her efforts to cure, abandons the case, the edges of the sore become hard, then cancerous, and death follows. Hence, disturb healing sores as little as possible; never pick off a scab, and yet what an intense desire there is on the part of a person having a sore to "pick" it. In all cases let the scab fall off of itself. It would be a good rule to never allow the finger to be introduced into the nose, because the parts are mostly gristle and have small vitality and very little healing power, while the sharp, hard nail is very apt to wound and re-wound. Nothing smaller that belongs to the hand than the ball of the thumb should be introduced into the nostril; the habit is unseemly, besides being dangerous; this should be taught children early, that it should not be done even in private; then there would be small danger of doing it before others; yet, unseemly as it is, it is done every day, adding thereunto the inspection thereof.

